# Regulation of Torpor in the Gray Mouse Lemur: Transcriptional and Translational Controls and Role of AMPK Signaling

**DOI:** 10.1016/j.gpb.2015.03.003

**Published:** 2015-06-17

**Authors:** Jing Zhang, Shannon N. Tessier, Kyle K. Biggar, Cheng-Wei Wu, Fabien Pifferi, Martine Perret, Kenneth B. Storey

**Affiliations:** 1 Institute of Biochemistry & Department of Biology, Carleton University, Ottawa, ON K1S 5B6, Canada; 2 Chemistry and Chemical Engineering Department, Royal Military College of Canada, Kingston, ON K7K 7B4, Canada; 3 Department of Surgery & Center for Engineering in Medicine, Massachusetts General Hospital & Harvard Medical School, Charlestown, MA 02129, USA; 4 Biochemistry Department, Schulich School of Medicine and Dentistry, Western University, London, ON N6A 5C1, Canada; 5 Department of Biology, Genetics Institute, University of Florida, Gainesville, FL 32611, USA; 6 UMR 7179 Centre National de la Recherche Scientifique, Muséum National d’Histoire Naturelle, Brunoy 91800, France

**Keywords:** Posttranslational modification, Histone H3, Ribosomal initiation factors, Metabolic rate depression, AMP-activated protein kinase

## Abstract

The gray mouse lemur (*Microcebus murinus*) is one of few primate species that is able to enter daily torpor or prolonged hibernation in response to environmental stresses. With an emerging significance to human health research, lemurs present an optimal model for exploring molecular adaptations that regulate primate hypometabolism. A fundamental challenge is how to effectively regulate energy expensive cellular processes (*e.g.*, transcription and translation) during transitions to/from torpor without disrupting cellular homeostasis. One such regulatory mechanism is reversible posttranslational modification of selected protein targets that offers fine cellular control without the energetic burden. This study investigates the role of phosphorylation and/or acetylation in regulating key factors involved in energy homeostasis (AMP-activated protein kinase, or AMPK, signaling pathway), mRNA translation (eukaryotic initiation factor 2α or eIF2α, eukaryotic initiation factor 4E or eIF4E, and initiation factor 4E binding protein or 4EBP), and gene transcription (histone H3) in six tissues of torpid and aroused gray mouse lemurs. Our results indicated selective tissue-specific changes of these regulatory proteins. The relative level of Thr172-phosphorylated AMPKα was significantly elevated in the heart but reduced in brown adipose tissue during daily torpor, as compared to the aroused lemurs, implicating the regulation of AMPK activity during daily torpor in these tissues. Interestingly, the levels of the phosphorylated eIFs were largely unaltered between aroused and torpid animals. Phosphorylation and acetylation of histone H3 were examined as a marker for transcriptional regulation. Compared to the aroused lemurs, level of Ser10-phosphorylated histone H3 decreased significantly in white adipose tissue during torpor, suggesting global suppression of gene transcription. However, a significant increase in acetyl-histone H3 in the heart of torpid lemurs indicated a possible stimulation of transcriptional activity of this tissue. Overall, our study demonstrates that AMPK signaling and posttranslational regulation of selected proteins may play crucial roles in the control of transcription/translation during daily torpor in mouse lemurs.

## Introduction

Daily torpor and multi-day torpor during seasonal hibernation are strategies used by a variety of mammalian species for fuel/energy conservation. By strongly suppressing overall metabolic rate and selectively shutting down various metabolic processes, animals can greatly extend the time that they can survive when facing environmental stress [1–3]. Exploration of the molecular mechanisms behind such fascinating phenomena not only provides answers to how natural torpor and hibernation are accomplished but also develops insights that could be applied for medical goals, including to avoid organ failure and to develop inducible human torpor as an aid to long term space flight [3–6]. The gray mouse lemur (*Microcebus murinus*) from Madagascar is one of the few primate species that is able to enter a hypometabolic state to utilize either daily torpor or multi-day hibernation [7]. Hence, this primate represents an ideal model for investigating torpor in the context of biomedical research.

The nocturnal gray mouse lemur enters torpor during its inactive period when ambient temperatures (*T*_a_) are low and arouses again to euthermic conditions via endogenous heat generation [8]. During daily torpor bouts, the body temperature (*T*_b_) of mouse lemurs can drop to 27–33 °C, whereas *T*_b_ falls to as low as 11.5 °C during 4 weeks of hibernation [9]. At the cellular level, one common theme of hypometabolism is the coordinated suppression of energy-expensive metabolic processes such as protein synthesis, cell proliferation, and growth, while upregulating pathways required for survival [10]. Studies have shown that various hibernating species achieve this through control at multiple levels including signal transduction, gene expression, and downstream biological processes [3,11].

In the context of the hypometabolic state, it is crucial to restrain net ATP expenditure, while effectively regulating molecular responses that are essential to survival. Reversible post-translational modification (PTM) of proteins/enzymes is one such regulatory mechanism for differential control and coordination of cellular processes. PTMs alter protein function by introducing structural changes through the addition or removal of covalently-attached functional groups on the amino acid chain. Reversible protein phosphorylation and protein acetylation are two well-studied PTMs. Major intermediary energy metabolism pathways rely heavily on the regulation of enzyme activity via reversible protein phosphorylation, so as many signal transduction pathways including those regulated by the AMP-activated protein kinase (AMPK) [12]. Reversible phosphorylation is also involved in transcriptional and translational control. For example, in association with acetylation, phosphorylation plays a crucial role in histone-mediated control of gene transcription [13] and phosphorylation events on key proteins are critical to the assembly of the eukaryotic translational complex that mediates protein synthesis [14].

AMPK-dependent signal transduction plays an important role in regulating energy-consuming biological processes when ATP availability is limited (*i.e.*, AMP levels are high) and AMPK is often considered as the cellular energy sensor. AMPK regulates cellular pathways to stimulate catabolic processes that improve ATP production while simultaneously inhibiting ATP-expensive anabolic activities. For example, AMPK-mediated phosphorylation pathway inhibits acetyl-CoA carboxylase that gates fatty acid synthesis [15]. Phosphorylation at Thr172 of its α-subunit [15] can trigger a ∼100-fold increase in kinase activity [16]. Therefore, the relative amount of phosphorylated AMPKα (p-AMPKα at Thr172) is a good indicator of AMPK activity.

Protein synthesis is an energy-expensive process that includes a stage-dependent assembly of various functional protein complexes [17]. For example, the multi-protein eIF4F pre-initiation complex brings mRNA to ribosomes if several components are accurately assembled. One of the components is eIF4E, which binds to the 5′m^7^G cap structure of a mature mRNA [18]. Such binding is negatively regulated by the eIF4E-binding protein (4EBP), which competes with the 5′ cap for binding with eIF4E, thereby inhibiting assembly of the pre-initiation complex. However, the inhibitory effect of 4EBP can be lifted by hyper-phosphorylation of the protein, causing the dissociation of 4EBP-eIF4E [14,18]. In addition, eIF4E is itself regulated by mitogen-activated protein kinases (MAPKs) induced phosphorylation at Ser209 [19,20]. Furthermore, protein synthesis is also regulated by eIF2, which is responsible for delivering the initiating Met residue to the assembling ribosome [21]. The α-subunit of eIF2 (eIF2) can be phosphorylated at Ser51 in response to environmental stresses, which attenuates the exchange of eIF2-GDP with eIF2-GTP, leading to translational inhibition.

Histones serve as central structural components of the nucleosome. Therefore, PTMs of histones play critical roles in controlling the interaction between *cis* and *trans* regulatory elements during initiation of gene transcription [22]. It is well-established that phosphorylation at Ser10 of histone H3 promotes gene transcription to facilitate a myriad of processes including growth and cell division [23,24]. Phosphorylation of histone H3 at Ser10 via stress-responsive kinase signaling pathways leads to a more open chromatin structure that allows gene transcription [25,26]. The N-terminus of histone H3 also houses multiple Lys residues that are subject to acetylation. Structural changes triggered by histone H3 acetylation lead to a loosening of the compact wrapping of DNA, thereby giving transcription factors and RNA polymerase access to the DNA-binding elements for transcription [27,28]. Furthermore, multiple studies have proposed a close relationship between histone H3 phosphorylation and acetylation [13].

Given that PTM is an effective and energy efficient way of modifying protein/enzyme functions, the current study utilized an enzyme-linked immunosorbent assay (ELISA) approach to analyze the levels of phosphorylated and/or acetylated key protein factors linked to AMPK signaling (AMPK), translation (eIF4E, 4EBP, and eIF2α), and transcription (histone H3). By comparing responses of different tissues from aroused versus torpid gray mouse lemurs, we explore the regulatory control of transcription and translation during torpor.

## Results and discussion

During mammalian torpor and hibernation, multiple energy-expensive processes are suppressed. For example, transcription, translation, and cell cycle progression are inhibited in organs of hibernating ground squirrels and hamsters [29–32]. Numerous studies suggest that signaling pathways upstream of crucial cellular processes are sensitive to limited energy availability and react accordingly to depress metabolic rate in a range of stress-tolerant species [33–36]. PTMs such as reversible protein phosphorylation frequently mediate survival adaptations under stress [10,37]. Therefore, the present study investigates the relative levels of posttranslationally-modified proteins involved in signal transduction, transcription, and translation processes using ELISA in lemur tissues comparing torpor and aroused states.

**Figure 1:**
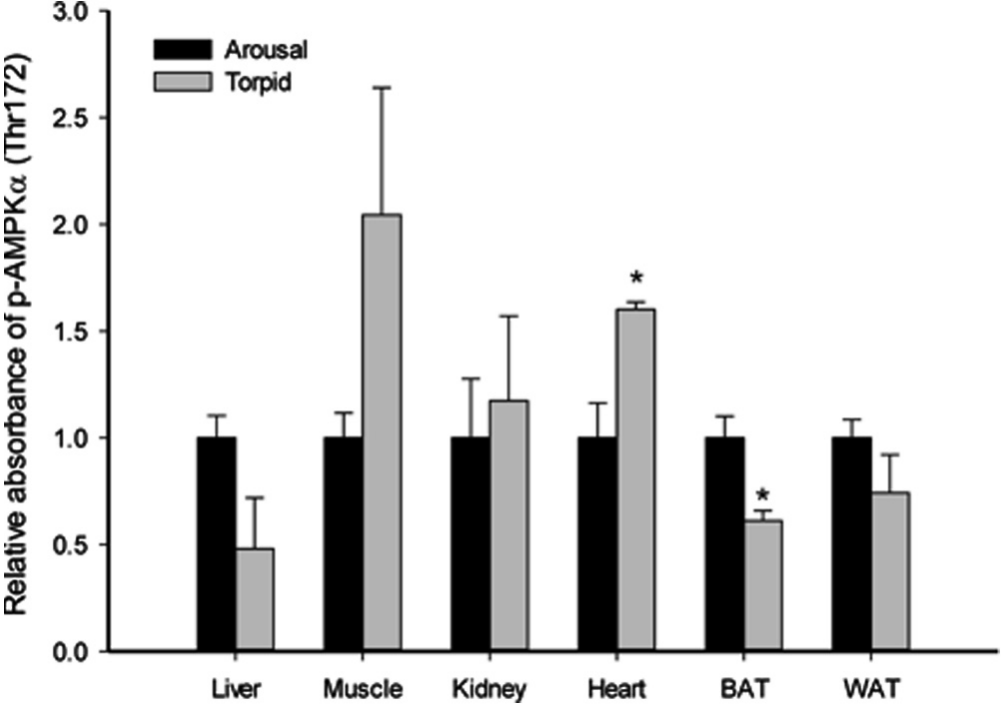
**Response of p-AMPKα (Thr172) to daily torpor in various lemur tissues** Histograms show relative absorbance based on antibody binding to the phosphoprotein target under control (aroused) and torpid states. Data are presented as mean ± SEM (*n* = 3–4 independent trials on tissue from different animals). ^*^Denotes significant difference from the corresponding control by the Student’s *t*-test (*P* < 0.05).

Differential responses of AMPK were previously reported for organs of hibernating ground squirrels [32]. To examine the response of the energy-sensing AMPK signaling pathway to daily torpor in gray mouse lemurs, we measured the relative level of p-AMPKα (Thr172) in torpid and aroused lemurs. Our results showed that in the heart tissue, the relative levels of p-AMPKα (Thr172) in torpid lemurs was significantly higher than that of aroused lemurs (1.6 ± 0.03-fold). On the other hand, p-AMPKα (Thr172) level in the BAT of torpid lemurs was only 61.1 ± 4.8% of that of the aroused lemurs, which is significantly lower (*P* < 0.05) (Figure 1). Since phosphorylation at Thr172 stimulates the catalytic activity of AMPK [15], these data suggest that AMPK was activated in the heart but inhibited in BAT during torpor. In the heart, AMPK signaling is closely related to fatty acid metabolism [38]. One potential action of AMPK-dependent stimulation of cardiac fatty acid metabolism is to promote fatty acid uptake via upregulation of the expression of the plasma membrane fatty acid transporter (FAT/CD36) and the associated intracellular fatty acid binding protein (FABP) [39]. FABPs are known to be upregulated in the heart and some other tissues of hibernating ground squirrels and bats [40,41]. Hence, the enhanced p-AMPKα (Thr172) levels observed in lemur heart may contribute to the stimulation of fatty acid uptake and transport to facilitate the use of lipids as fuels during torpor.

AMPK signaling also influences mRNA translation. One mechanism is via its effects on the target of rapamycin (TOR)-4EBP pathway during translation initiation [42,43]. Active AMPK inhibits TOR (often called mTOR in mammals) activity through direct phosphorylation of TOR, as well as the TOR inhibitor protein, tuberous sclerosis (TSC) complex TSC1-TSC2 [44,45]. Increased p-AMPKα (Thr172) level in the heart of torpid lemurs (signaling energy limitation) correlates with lowered metabolic rate and is an indicator of inhibitory regulation of energy-expensive protein synthesis. However, the level of p-AMPKα dropped significantly in BAT of the torpid lemurs. Given that BAT is responsible for non-shivering thermogenesis that drives the rewarming of the body during arousal [46], it is possible that BAT must retain some level of protein synthesis activity in the hypometabolic state, which may explain the observed suppression of AMPK in this tissue. Indeed, analysis of protein synthesis rates in an *in vitro* translational assay revealed no change in ^3^H-leucine incorporation into protein in BAT comparing hibernating ground squirrels to euthermic ones, whereas the rate in kidney extracts of hibernating squirrels was only 15% of the euthermic ones [45]. Furthermore, no signs of AMPK activation were observed in BAT of ground squirrels during hibernation [32,47]. Overall, these data suggest that the gray mouse lemur and 13-lined ground squirrel may share a similar strategy of BAT adaptation under torpid conditions, to maintain a state of readiness for protein synthesis that would support immediate demands by the tissue whenever thermogenesis is initiated.

**Figure 2:**
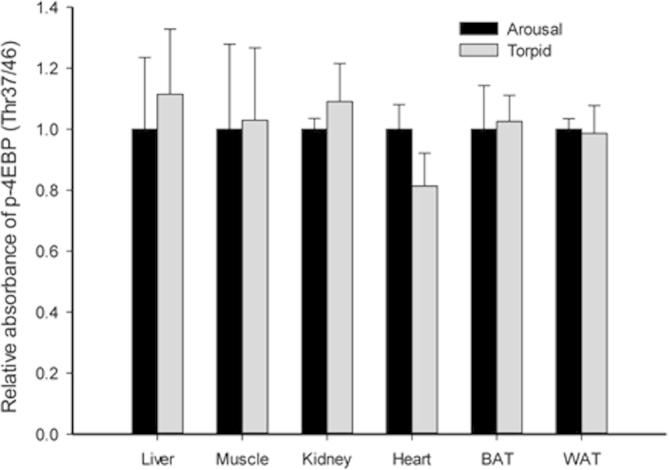
**Response of p-4EBP (Thr37/46) to daily torpor in various lemur tissues** Histograms show relative absorbance based on antibody binding to the phosphoprotein target under control (aroused) and torpid states. Data are presented as mean ± SEM (*n* = 3–4 independent trials on tissue from different animals). ^*^Denotes significant difference from the corresponding control by the Student’s *t*-test (*P* < 0.05).

**Figure 3:**
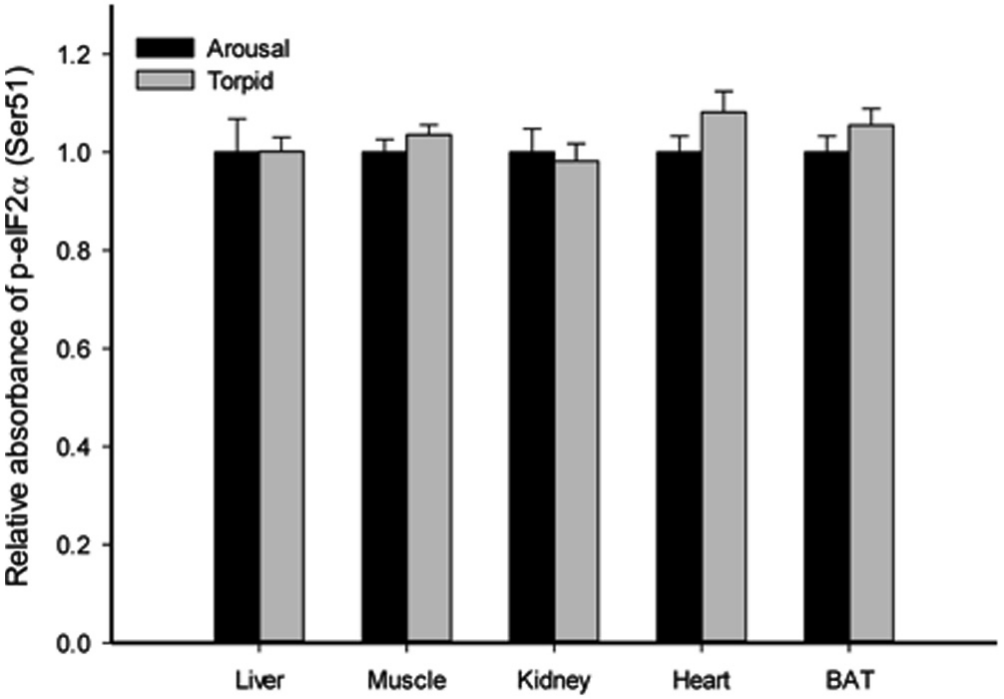
**Response of p-eIF2α (Ser51) to daily torpor in various lemur tissues** Histograms show relative absorbance based on antibody binding to the phosphoprotein target under control (aroused) and torpid states. Data are presented as mean ± SEM (*n* = 3–4 independent trials on tissue from different animals). ^*^Denotes significant difference from the corresponding control by the Student’s *t*-test (*P* < 0.05).

The effects of torpor on other components of the TOR-4EBP-eIF4E axis were also investigated in lemur tissues. Active TOR phosphorylates 4EBP to release inhibitory binding of eIF4E and thereby promote the assembly of the pre-translation initiation complex [43]. Our results showed no significant changes in the p-4EBP level in any of the tissues tested (Figure 2). Unaffected p-4EBP levels suggest that a potential AMPK-dependent control over translation may be exerted at other stages of translation. Similarly, the p-eIF2α level in torpid lemurs remained comparable to that in aroused animals in all tissues tested (Figure 3). Since AMPK is also able to regulate mRNA translation through eukaryotic elongation factor 2 (eEF2) [42], it is possible that the AMPK signaling exerts regulation over translation in lemur heart and BAT at another stage of translation, such as elongation. Indeed, elevated p-eEF2 (Thr56) levels were observed in liver, WAT, and brain of hibernating ground squirrels, supporting the inhibitory control at this level [32].

**Figure 4:**
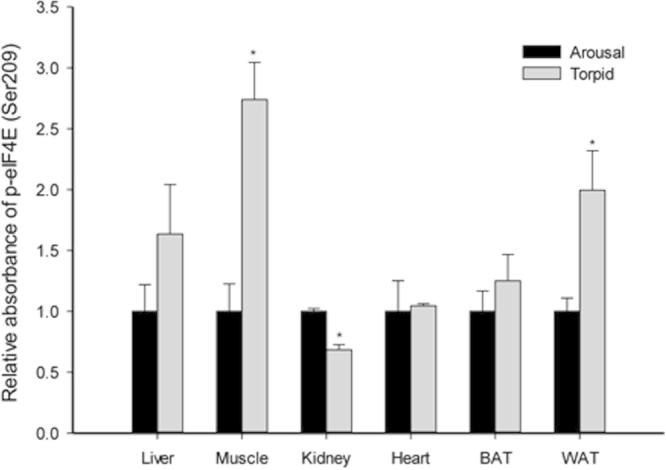
**Response of p-eIF4E (Ser209) to daily torpor in various lemur tissues** Histograms show relative absorbance based on antibody binding to the phosphoprotein target under control (aroused) and torpid states. Data are presented as mean ± SEM (*n* = 3–4 independent trials on tissue from different animals). ^*^Denotes significant difference from the corresponding control by the Student’s *t*-test (*P* < 0.05).

Interestingly, the level of p-eIF4E (Ser209) showed a tissue-specific response to daily torpor. Skeletal muscle and WAT possessed significantly more p-eIF4E (2.74 ± 0.3 and 2.0 ± 0.3 fold when compared to the arousal level, respectively; *P* < 0.05); whereas kidney showed a decrease to 68.4 ± 4% of the level observed in the aroused animals (Figure 4). In addition to being a major downstream target to AMPK signaling, eIF4E is also regulated by other stress-responsive kinase pathways, such as MAPKs. For example, eIF4E can be phosphorylated at Ser209 by p38MAPK and ERK [19,20]. Given that p38MAPK and ERK pathways respond to distinct stimuli, extracellular stresses [48–50], and mitogenic stimuli [51], respectively, the Ser209 phosphorylation events on eIF4E from those pathways make translation capable of responding to various extracellular conditions. Although further studies are needed in order to fully elucidate the function of Ser209 phosphorylation on eIF4E [19], the tissue-specific changes observed in the current study suggest that p38MAPK and/or ERK signaling pathways might contribute to control of the daily torpor in lemurs. Indeed, the protein levels of both p38MAPK and ERK increased significantly in skeletal muscle of torpid lemurs, which is consistent with the observed enhancement of phosphorylated eIF4E content in the present study [52]. Activation of p38MAPK in skeletal muscle has also been reported in Richardson’s ground squirrels and little brown bats [53,54], indicating that p38MAPK is sensitive to torpor conditions in multiple hibernating species. Similarly, increased total protein and phosphorylation levels of major kinases in MAPK cascades were observed in WAT of torpid lemurs [52]. Since WAT is the main storage organ for lipid, the activation of MAPK cascades may play crucial roles in the lipid-based energy metabolism that supports hibernation [55]. Biggar et al. [52] also demonstrated signs of inhibition of p38MAPK and ERK pathways in lemur kidney during daily torpor (decreased p-p38MAPK and p-ERK levels), which agrees with our observation that kidney p-eIF4E content dropped significantly during torpor.

**Figure 5:**
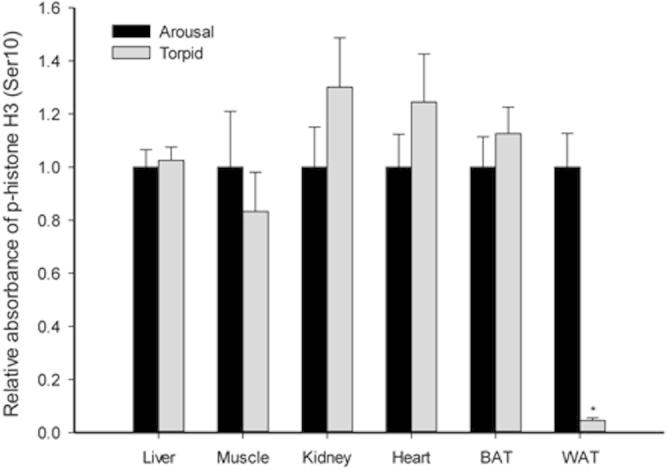
**Response of p-histone H3 (Ser 10) to daily torpor in various lemur tissues** Histograms show relative absorbance based on antibody binding to the phosphoprotein target under control (aroused) and torpid states. Data are presented as mean ± SEM (*n* = 3–4 independent trials on tissue from different animals). ^*^Denotes significant difference from the corresponding control by the Student’s *t*-test (*P* < 0.05).

**Figure 6:**
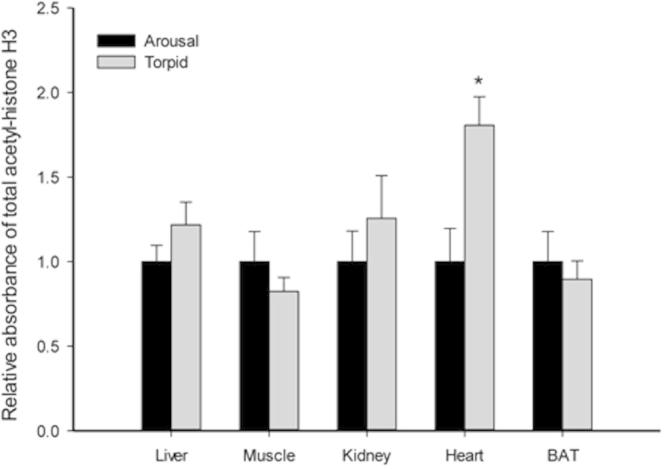
**Response of total acetyl-histone H3 to daily torpor in various lemur tissues** Histograms show relative absorbance based on antibody binding to the acetyl-protein target under control (aroused) and torpid states. Data are presented as mean ± SEM (*n* = 3–4 independent trials on tissue from different animals). ^*^Denotes significant difference from the corresponding control by the Student’s *t*-test (*P* < 0.05).

Gene transcription is another ATP-expensive process in cells that typically shows global suppression during hypometabolism [56]. Posttranslational phosphorylation and acetylation of histone H3 lead to transcriptional activation by opening up chromatin structure to facilitate binding of the transcriptional apparatus [25]. We thus analyzed levels of p-Histone H3 (Ser10) and acetylated histone H3 in multiple tissues of lemurs. The results show that both p-histone H3 (Ser10) and acetyl-histone H3 levels remained unchanged in liver, skeletal muscle, kidney, and BAT during torpor (Figure 5 and Figure 6). However, WAT exhibited a strong and significant reduction in the phosphorylated histone H3 level during torpor to only 4.5 ± 1% of the corresponding arousal level (Figure 5; *P* < 0.05), suggesting suppressed transcriptional activity in this tissue during torpor. On the other hand, the heart was the only organ to show a significant change in acetylated histone H3 levels in torpid lemurs, which is 1.8 ± 0.2-fold of the control animals (Figure 6; *P* < 0.05), which could indicate a stimulation of transcription activity during torpor. The heart must remain relatively active (although at a much lower heart beat rate) during torpor in order to circulate blood and maintain oxygen and substrate supplies to tissues [57]. Thus, the observed increase in acetylated histone H3 levels may facilitate a more flexible chromosome structure needed to support selective gene expression during torpor. Indeed, previous studies have reported substantial torpor-induced gene expression in the heart during ground squirrel hibernation [58–60]. Our observations on the heart acetyl-histone H3 level indicate a similar pattern in lemur during daily torpor.

## Conclusion

Accordingly, the current study provides insights into the molecular regulatory network supporting daily torpor in gray mouse lemurs stretching from the energy sensing AMPK pathway to specific downstream processes including gene transcription and mRNA translation. While the relative levels of most posttranslationally-modified targets were unchanged during torpor as compared with the aroused control, some tissue-specific changes were observed. For example, p-AMPKα (Thr172) levels increased in the heart but decreased in BAT. Since some organs such as BAT serve unique functions that are crucial for survival during torpor, it is not surprising that tissue-specific changes in PTM-dependent functional regulation of proteins were seen under torpor conditions. These findings are consistent with the conservation and re-prioritization of ATP expenditures in the hypometabolic state. This study demonstrates for the first time that posttranslational modifications play a role in the regulation of transcription/translation and energy homeostasis (via AMPK signaling) during gray mouse lemur daily torpor. Our work provides a solid foundation for future studies aimed at fully depicting the molecular signatures of daily torpor, ranging from high throughput transcriptomic and proteomic studies, to the potential species specific genome characterizing.

## Materials and methods

### Animal care

Animal care and experiments are described in detail by Biggar and his colleagues [52]. All imported tissues were logged according to the Convention on International Trade in Endangered Species of Wild Fauna and Flora (CITES) regulations (import permit No: 10cA02291/QWH and export permit No: FR1009118231-E). All tissues were stored at −80 °C prior to use.

### Protein extraction

Frozen tissue samples (up to 50 mg) were homogenized 1:4 (w/v) in a pre-chilled lysis buffer (Millipore, catalog No. 43-040) with 1 mM Na_3_VO_4_, 10 mM ß-glycerophosphate and 1% protease inhibitor cocktail (catalog No. PIC001, BioShop) added using a Dounce homogenizer. Samples were incubated on ice for 30 min before centrifugation at 12,000 × *g* for 20 min at 4 °C. Supernatants were collected as total soluble protein lysates and protein concentrations were determined using the Bradford assay. Aliquots of the lysate were then adjusted to a final working concentration of 0.7 μg/μl using the assay buffer provided with the corresponding ELISA kit. For each assay, the amount of protein added was optimized for each tissue and kit, ranging from 5 to 60 μg.

### ELISA

PathScan ELISA kits (New England Biolabs, Canada) were used to assess the amount of posttranslationally-modified 4EBP (p-4EBP Thr37/46, catalog No. 7216S), eIF4E (p-eIF4E Ser209, catalog No. 7938S), eIF2α (p-eIF2α Ser51, catalog No. 7286S), histone H3 phosphorylation (p-histone Ser10, catalog No 7155S), histone H3 acetylation (pan A-histone H3, catalog No. 7232S) and AMPKα (p-AMPKα Thr172, catalog No. 7959C) in the liver, skeletal muscle, heart, kidney, BAT, and WAT. Assays were carried out according to manufacturer’s instructions. Briefly, capture antibody-coated microwell strips were equilibrated to room temperature. Protein samples were diluted to a value appropriate for each kit using sample diluent (supplied with the ELISA kits). Aliquots of 100 μl of diluted protein samples were added into wells of the microwell strips (mounted in a 96 well microplate frame), which were subsequently covered with sealing tape and allowed to incubate for 2 h at 37 °C. All wells were washed three times with diluted (1 ×) wash buffer provided with the kit. Then 100 μl of the detection antibody was added into each well and incubated with samples at 37 °C for 1 h. After washing as above, samples were then incubated with 100 μl HRP-conjugated secondary antibodies for 30 min at 37 °C. Secondary antibodies were discarded after incubation and the sample wells were washed before adding 100 μl tetramethylbenzidine (TMB) substrate for colorimetric detection. After 10 min, 100 μl aliquots of stop solution were added into the sample wells and absorbance was read and quantified at 450 nm using a spectrophotometer.

### Data analysis

The quantified signals were compared between aroused and torpid animals. The results are presented as relative absorbance. All data are expressed as mean ± SEM (*n* = 3–4 individual animals). The Student’s *t*-test was employed to assess differences between samples from aroused and torpid animals and difference was considered significant with *P* < 0.05.

## Authors’ contributions

All authors contributed to the conception and design of the project and to the editing of the manuscript. MP and FP carried out the animal experiments; JZ, SNT, KKB, and CWW conducted biochemical assays. Data analysis and assembly of the draft manuscript was carried out by JZ, SNT, and KBS. All authors read and approved the final manuscript.

## Competing interests

The authors have declared no competing interests.
